# A three-year dataset supporting research on building energy management and occupancy analytics

**DOI:** 10.1038/s41597-022-01257-x

**Published:** 2022-04-05

**Authors:** Na Luo, Zhe Wang, David Blum, Christopher Weyandt, Norman Bourassa, Mary Ann Piette, Tianzhen Hong

**Affiliations:** 1 Lawrence Berkeley National Laboratory, Berkeley, California 94720 United States; 2 Department of Civil and Environmental Engineering, The Hong Kong University of Science and Technology, Hong Kong SAR, China

**Keywords:** Mechanical engineering, Energy modelling

## Abstract

This paper presents the curation of a monitored dataset from an office building constructed in 2015 in Berkeley, California. The dataset includes whole-building and end-use energy consumption, HVAC system operating conditions, indoor and outdoor environmental parameters, as well as occupant counts. The data were collected during a period of three years from more than 300 sensors and meters on two office floors (each 2,325 m^2^) of the building. A three-step data curation strategy is applied to transform the raw data into research-grade data: (1) cleaning the raw data to detect and adjust the outlier values and fill the data gaps; (2) creating the metadata model of the building systems and data points using the Brick schema; and (3) representing the metadata of the dataset using a semantic JSON schema. This dataset can be used in various applications—building energy benchmarking, load shape analysis, energy prediction, occupancy prediction and analytics, and HVAC controls—to improve the understanding and efficiency of building operations for reducing energy use, energy costs, and carbon emissions.

## Abstract (summary)

**Table:** 

Measurement(s)	indoor temperature • Electricity • Indoor occupancy
Technology Type(s)	temperature sensor • electricity use sensor • occupancy sensor
Factor Type(s)	building energy management • HVAC operation
Sample Characteristic - Environment	office building
Sample Characteristic - Location	United States of America

## Background & Summary

Buildings consume approximately 40% of the primary energy in the United States^1^ and about one-third globally. Today’s technologies (e.g., energy efficiency, sensors, and advanced controls) could reduce energy use in buildings by up to 50%^2^. Reducing energy waste in buildings and optimizing building operations require access to a diverse and integrated set of data^3,4^. However, it is currently time consuming and hard to find datasets that have adequate data coverage (e.g., indoor and outdoor environmental parameters, occupant parameters, energy end uses, building system operational parameters), good data quality, and clear documentation (e.g., metadata description).

Measuring ground truth at high resolution in all buildings is impractical and challenging^5,6^. Therefore, it is critical to collect, curate, and make publicly available high-resolution data from a small number of buildings that have broad applicability to a variety of high-impact use cases. Such datasets can provide a common, high-quality benchmark against which competing algorithms can be fairly compared.

The great majority of energy in residential and commercial buildings is used to deliver services for occupants^7^. Numerous studies emphasize the role that occupants play in influencing energy consumption in buildings^8,9^. An accurate prediction of occupant counts can largely improve building energy efficiency through demand flexibility control (DFC)^10^ and model predictive control (MPC)^11^. Despite the significance of occupant information, data collection is still challenging due to cost and privacy concerns.

In 2020, an unprecedented global lockdown was enforced to control the spread of COVID-19 in many countries. The impact of pandemic lockdown on building energy use is complicated due to different building types, climate conditions, and control and operating policies. The restriction on occupants’ activities tended to reduce energy consumption in office buildings, particularly in electric devices such as lighting and plug loads^12^. However, the lingering effects of the lockdown may hamper the goal of improving building energy efficiency by adding uncertainty and additional requirements to minimize the spread of the virus^13^. Therefore, it is critical to identify how the COVID-19 pandemic is influencing building and system operations, and to understand building energy use and efficiency in these times.

This paper presents the curation and development of a building performance dataset and related metadata semantic models of the building and systems. The uniqueness of this dataset includes:

- A rich high-resolution three-year time-interval data of a real office building, which includes two years of pre-pandemic data, and the year of 2020 when the COVID-19 pandemic started.
- The building was used for model predictive control research and field testing.
- The dataset has camera-based occupant count measurements as well as proxy virtual sensing from the WiFi-connected device count.
- A Brick model^14^, which is an open-source effort to standardize semantic descriptions of the physical, logical, and virtual assets in buildings and the relationships between them, was developed to represent the metadata of the sensors, meters, and HVAC systems.
- A semantic description of the dataset (including building and system characteristics, and information on data curation, data quality, data categories, and application aspects) was developed.

The dataset can be used to support various use cases, including:

- Building energy benchmarking at the whole-building and end-use levels to understand relative energy efficiency compared with peer buildings (same use type, same climate zone) and improvement opportunities to reduce energy use^15–17^.
- Load shape analysis to understand whole-building and end-use level demand profiles^18^.
- Building energy prediction using statistical or machine learning algorithms^19^.
- Occupancy analytics to understand occupancy patterns and correlation between occupancy level and building energy use^20^.
- Development and validation of building thermal simulation models for use in model predictive control^21^.
- Fault detection and diagnostics to identify HVAC operational issues^22^.
- Prediction and validation of occupant count using WiFi connected device count^23^.

## Methods

### Description of the building and systems

#### Building

**Fig. 1:**
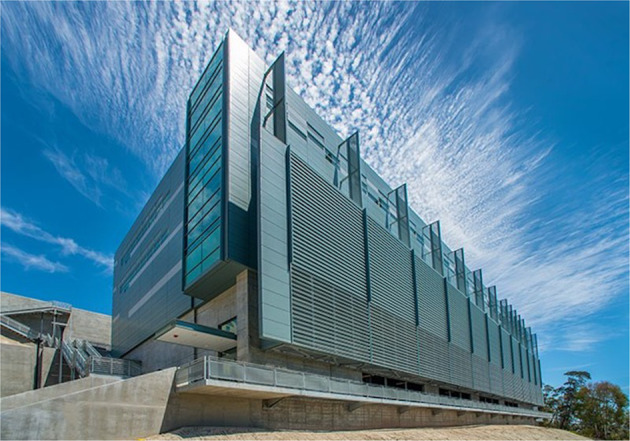
The office building in Berkeley, California.

The target building (Fig. 1) is a medium-sized office building (i.e., Building 59 or Wang Hall) located inside the Lawrence Berkeley National Laboratory (Berkeley Lab) campus in Berkeley, California. The building has 10,400 m^2^ of conditioned spaces on four floors. The lower level provides space for mechanical systems, the second level is the National Energy Research Scientific Computing Center (NERSC), and the third and fourth levels are office spaces. The ground office floor (third floor) is primarily closed office space, while the second office floor (fourth floor) is primarily open office space.

The building structure is steel-framed with an exterior metal curtain wall system with integrated windows and foamed insulation core. There are vertical sunshades on the exterior. In office areas, finished floors with carpeting are raised above structural concrete slabs, creating the plenum for the underfloor air distribution (UFAD) HVAC system. R30 insulation is added between the bottom of the ground office floor and the top of the high performance computing area, while a dropped ceiling plenum separates the ground level office area from the second level office area as well as the second level office area from the roof. The roof is a white single ply PVC roofing membrane over ½” cover board and insulation layers on a concrete roof deck.

**Fig. 2:**
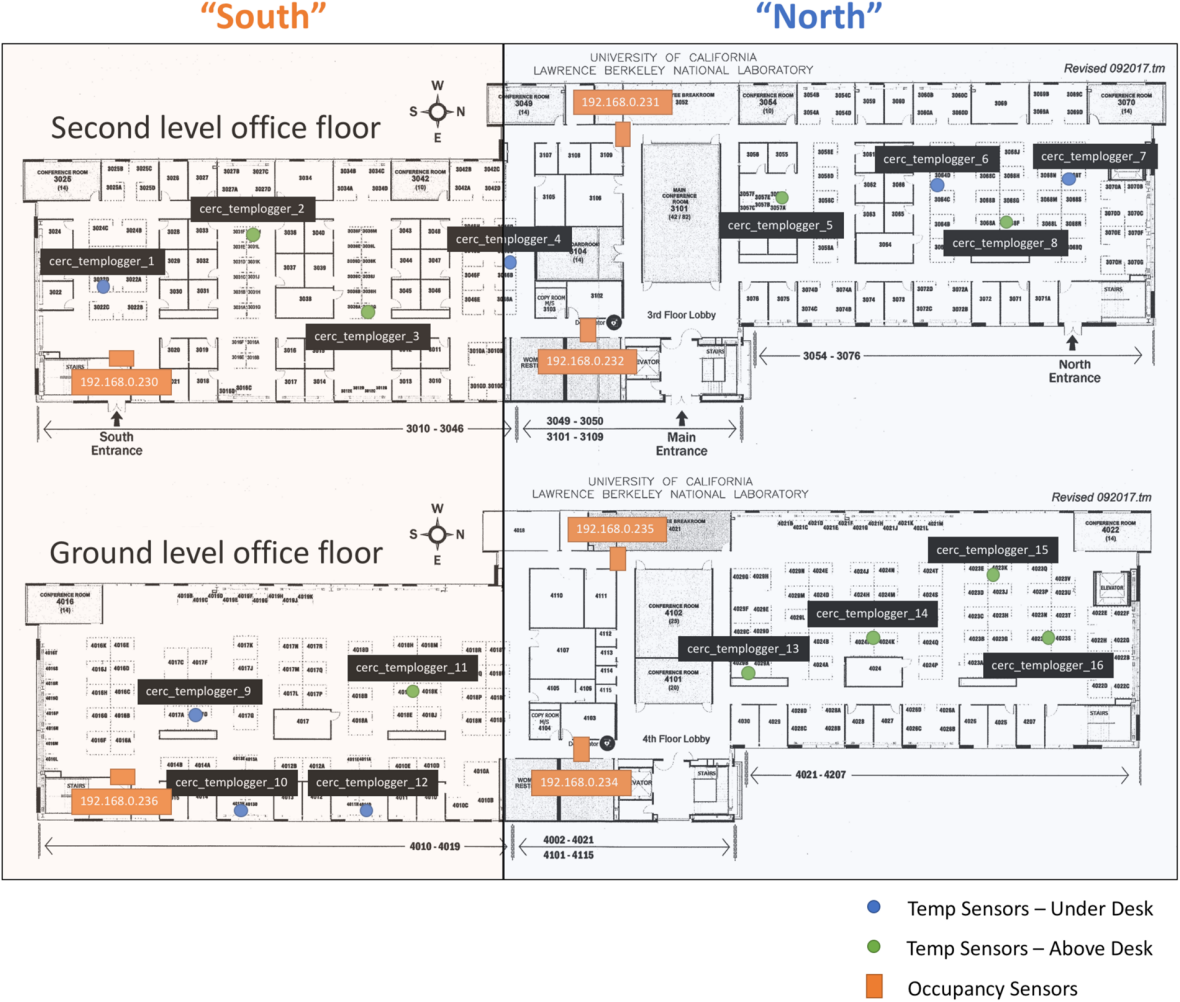
Location of temperature sensors and occupant sensors.

The building is divided into 57 thermal zones. Thermal zones with exterior walls and windows are classified as exterior zones; others are classified as interior zones. The temperatures of exterior zones are measured by the wall-mounted sensors installed within each zone served by an under-floor terminal (UFT) as part of the building automation system (BAS). The temperatures of interior zones are measured by 16 sensors that were added by the research team at desk level, which are built with Raspberry Pi Zero W and DS18B20 Digital Temperature Sensors. These temperature sensors are located as close as possible to where occupants stay, for instance, at their workstations. In addition, to measure occupant counts, we deployed camera-based sensors manufactured by TRAF-SYS at the six entrances/exits of the southern wing of the building. Figure 2 presents the locations of temperature sensors and occupant sensors.

#### HVAC Systems

**Fig. 3:**
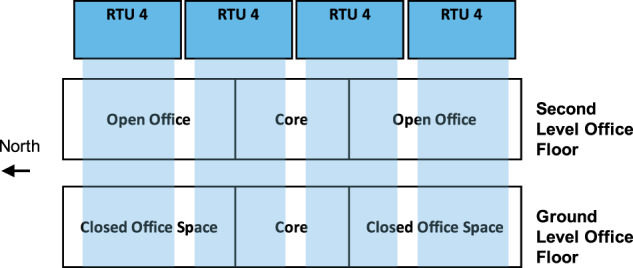
Elevation schematic of RTU service coverage of the office levels.

Heating and cooling are provided to the offices by a UFAD system. The system uses four roof-top units (RTUs) located on the roof with water-cooled direct expansion (DX) coils to supply cool air to the underfloor plenums. Each RTU serves the ground level and second level offices between particular column lines of the building, as depicted in Fig. 3, though the areas of service are not separated by internal wall partitions. The four RTUs operate their supply fans at the same speed, instead of separately controlling to their own sensors and setpoint. The thermal zones served by each RTU are summarized in Table 1. The design airflow of each RTU is 33,980 m^3^/hr (20,000 cubic feet per minute or cfm), with minimum outdoor air of 8,495 m^3^/hr (5,000 cfm). The supply fan motor is 20 horsepower (HP) (16.4 brake horsepower or BHP) and the return fan motor is 7.5 HP (3.7 BHP), each equipped with variable speed drives. The cooling capacity of each RTU is 356 MBH (30 tons or 105.5 kW) with two 13-HP R410A scroll compressors. Submittals indicate requirements for variable speed control of each compressor from 10% to 100%. There are 50 fan-powered terminal units (UFTs) with hydronic heating coils to provide reheat. Air from the RTU is supplied to the underfloor plenum and then delivered to interior and exterior zones directly through floor diffusers and additionally to exterior zones through fan-powered UFTs. The UFTs reheat this perimeter air if necessary. The condenser water from the RTUs is cooled by heat exchangers connected to the induced draft crossflow cooling towers located next to the building on the mechanical level. These cooling towers are shared with the high performance computing (HPC) cooling equipment, which dominate the load on the cooling towers. UFT heating is produced by a 117 kW (400 MBH) (nominal) heat pump (air-source type before March 2019, later replaced with water-source) located on the mechanical level of the building and two 3 HP variable frequency drive (VFD) pumps.

**Table 1:** Key Electrical Panels.

Lighting zone	RTU	Thermal zones
North Wing	1	36, 37, 38, 39, 40, 41, 42, 64, 65, 66, 67, 68, 69, 70
North Wing	2	19, 20, 27, 28, 29, 30, 31, 32, 33, 34, 35, 43, 44, 49, 50, 57, 58, 59, 60, 62, 63, 71, 72
South Wing	3	18, 25, 26, 45, 48, 55, 56, 61
South Wing	4	16, 17, 21, 22, 23, 24, 46, 47, 51, 52, 53, 54

**Fig. 4:**
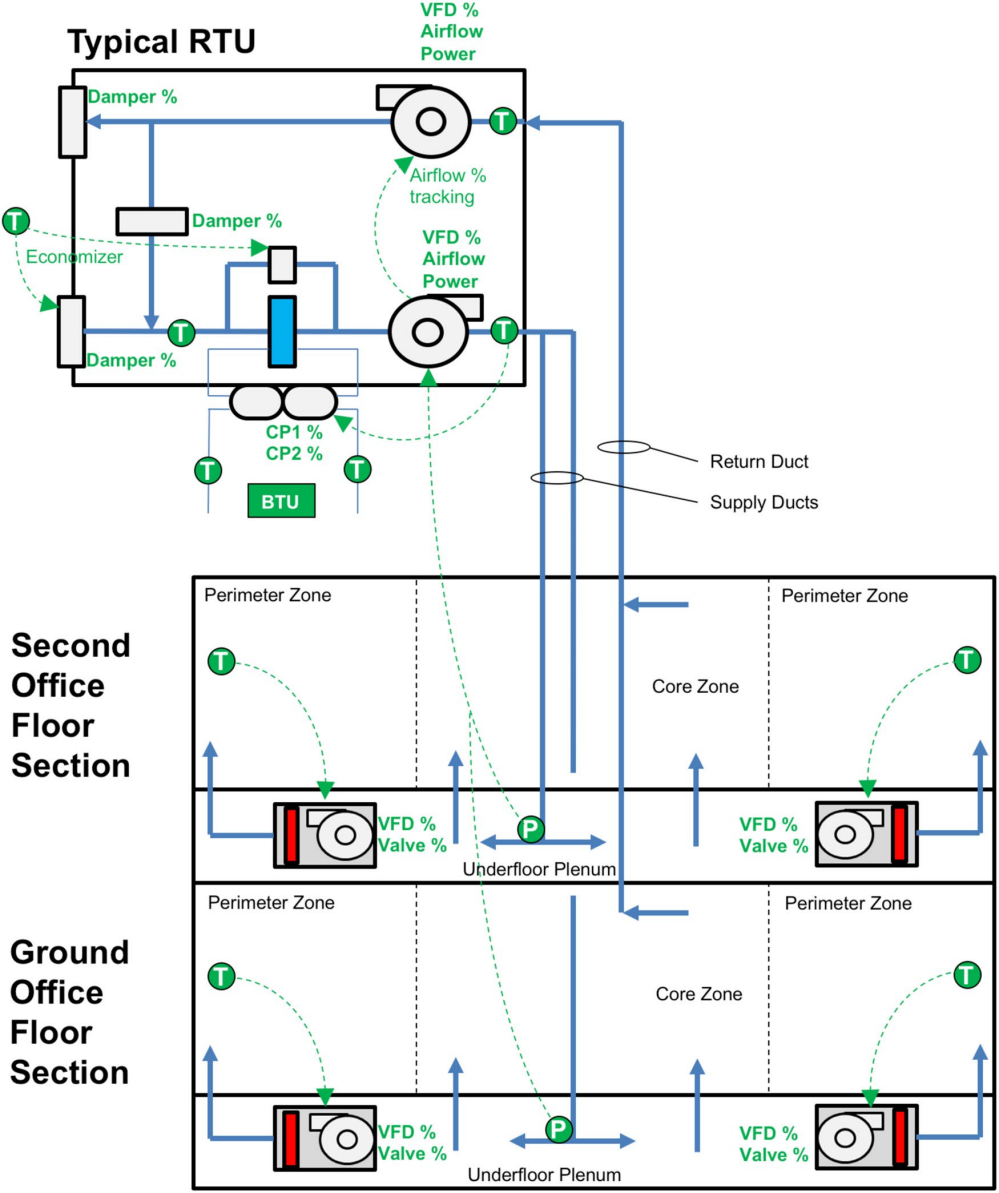
Control schematic for the RTU HVAC systems. Important temperature (T) and pressure (P) sensors and associated control points available through the ALC BMS interface are labelled.

HVAC systems are controlled by an Automated Logic (ALC) WebCTRL building management system (BMS) (Automated Logic 2017) with an extensive array of sensors. BMS sensors and controllers are networked to a NERSC network firewall protected server hosted within the Building 59 computer room facility. Read access to the ALC BMS logic and data trends is provided through a web-hosted graphical user interface (GUI). Figure 4 shows the typical control schematic for each RTU.

During the data collection periods (2018–2020), two control modes were applied: conventional rule-based control (RBC) and model predictive control (MPC). The starting and ending dates of the MPC testing are listed in Table 2. RBC used a predetermined zone temperature setback schedule (second floor office, Saturdays) to select the temperature setpoint of each UFT as well as the minimum outside air flow damper position. In late summer 2020, more functions were introduced to the RBC control, including a fresh air flow rate setpoint and a smoke mitigation mode for wildfire season, which, when enabled, would close the outside air damper to a minimum to prevent economizer operation. MPC adopted an optimization-based approach to determine the optimal setpoints for RTU supply air temperature and fan speed based on the current states and predicted disturbances. Local controllers continued to track their set points using the pre-existing RBC, except for the fan speed controller. MPC mode was on in the fall and winter of 2020, which will be further illustrated in Table 2.

**Table 2:** Key timeline of events for the building and data collection.

Event number	Starting date	Ending date	Event
1	2018/11/12	2018/11/20	Wildfire
2	2020/03/18	2020/12/31	Shelter-in-place
3	2020/08/24	2020/09/06	Wildfire
4	2020/10/20	2020/10/27	MPC testing
5	2020/11/02	2020/11/06	MPC testing
6	2020/11/13	2020/11/19	MPC testing
7	2020/12/04	2020/12/14	MPC testing

#### Electrical systems

Two transformers feed the building’s office and HVAC main switchboards. The office main switchboard (4000A, 277/480V) serves lighting and receptacle panels on the ground and second level office floors, along with other panels for common areas, special rooms, and emergency power. The HVAC main switchboard (4000A, 277/480V) serves mechanical equipment throughout the building. Table 3 summarizes the services provided by six key electrical panels.

**Table 3:** Key Electrical Panels.

Panel Label	Service
590A1A	North Office Lighting with Compute Lighting
590A15A	South Office Lighting with Compute Lighting
590A2A	North Plug Loads
590A14A	South Plug Loads
596A1A1A	RTU 3–4 (North) with Elevator
596A1A2A	RTU 1–2 (South) with Elevator

Electrical systems are metered at the panel level by NERSC Center for purposes of data center energy consumption monitoring and benchmarking. The two main plug load panels, two main lighting panels, and two main HVAC panels are metered using General Electric Trip units. The two plug and lighting panels serve the north and south wing of the two office floors (e.g., one panel serves one wing of both floors), while the two HVAC panels contain the RTU units, two on each, as well as the building elevators. Electrical meter data are accessible through a Grafana (Grafana Labs 2017) web-hosted GUI and can be downloaded into CSV files.

#### Lighting systems

The lighting system in the offices is composed of Philips T8 32W fluorescent light fixtures, occupancy/vacancy sensors, photocell light detectors, and a dedicated lighting control server with workstation. The light fixtures are controllable through a Quantum Light management hub with Ecosystem Energi Savr Nodes. The photocell light detectors are placed in perimeter zones, while occupancy sensors are placed throughout the office zones. The occupancy sensors are used to control the on/off state of lights in a lighting zone. There are manually controlled roller shades on the inside of windows serving office and meeting spaces. Lighting systems are metered through the Lutron Quantum Vue (Lutron 2017) browser-based GUI. Energy consumption data of each lighting zone can be downloaded via the GUI into CSV files.

### Data collection

**Fig. 5:**
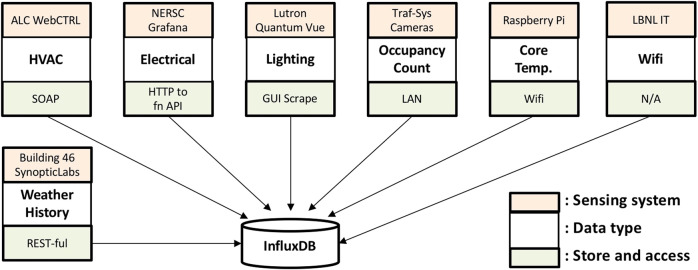
Data collection systems and the central influxDB database.

Data from Building 59 comes from various sources and systems. Figure 5 shows the group of data points and their collection systems. To store the data in a central database, all the data streams are pulled from their sources and systems and integrated into an influxdb database, an open source time series database. In this way, the data collection from different data sources is independent: i.e., the crash of one data source will not influence others.

For HVAC systems operational data, the ALC SOAP web interface is used to retrieve data of specified points from the ALC WebCTRL Building Automation System. For the electrical consumption data, the ElasticSearch database, on which the data is held for specific points through a web endpoint, is queried. For the site weather data, HTTP RESTful requests are made to the Synopticlabs web application programming interface (API) to gain access to data from the weather station located on the Berkeley Lab campus.

In addition to the existing BAS data points, occupant sensors and indoor air quality (IAQ) sensors were installed in the building. Camera-based occupancy sensors were deployed to collect occupant count data. The camera-based sensor can detect the number of people entering and leaving the space. Integrating the net flow of people entering the border can inform the number of occupants in the target area. The sensor accuracy was validated by sending a crew of researchers to manually count the net number of people through each entrance. Additionally, Wi-Fi data were collected with the help of Berkeley Lab’s IT department. The total number of connected devices at each Wi-Fi Access Point (AP) were collected and aggregated at the floor level based on the location of each AP. The Wi-Fi connection counts could serve as a proxy variable of occupant counts.

There are 16 air temperature sensors built with Raspberry Pi Zero W and DS18B20 Digital Temperature Sensors. The Raspberry Pis are running Raspbian Lite (no GUI) and are connected to a power source. The communication is done through Berkeley Lab’s WiFi network: the Raspberry Pis push the measured indoor air temperature to the database every 10 minutes. Temperature sensors are located as close as possible to where occupants stay, for instance, at their workstations.

Historical weather data for the building are available through SynopticLabs (MesoWest and SynopticLabs 2017) from a tower-mounted weather station located at the Berkeley Lab’s campus, approximately 300 meters northeast of the building. Measurements include outdoor air temperature, dew point, precipitation, pressure, relative humidity, solar irradiation, wind speed, and wind direction. The measurement timestep is 15 minutes. Data can be downloaded from a web-hosted GUI into CSV files or through the SynopticLabs RESTful HTTP API.

#### Key timeline of building operational changes and data collection

There are operational changes to the building during the three-year data collection period. Table 2 lists seven major building operational changes, which can be categorized into three types: wildfire, shelter-in-place due to the COVID-19 pandemic, and MPC testing to enhance building operation efficiency. In about three weeks during 2018 and 2020, the building closed the outdoor air dampers to minimize outdoor air flow rate due to air pollution caused by the wildfires. In late March 2020, most staff in the building started working from home, which significantly reduced the occupancy of the building. In about five weeks during 2020, MPC was tested in the building to optimize the operation of the HVAC systems to reduce energy use.

### Data curation

#### Data curation workflow

**Fig. 6:**
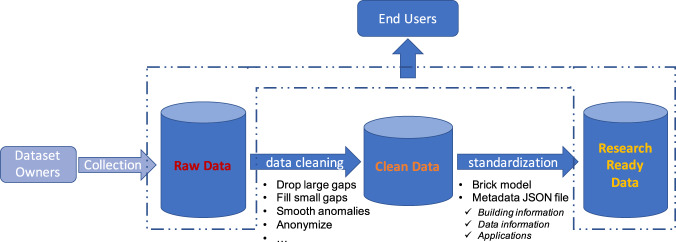
Diagram of the dataset curation workflow.

The cleaning and curation process of the raw dataset follows the proposed workflow as shown in Fig. 6. First, the data are cleaned to generate a clean version of the time-series data. This process includes identifying and dropping large gaps, filling small gaps using multiple interpolation algorithms based on the gap size, smoothing anomalous values, as well as anonymization if necessary. Then, two metadata models/files are generated to describe the semantic information of the building assets and data points at different levels of detail. More granularly, the *Brick model* provides semantic information of the physical, logical, and virtual assets, as well as their relationships in buildings; the self-documented *Metadata JSON file* helps streamline data sharing and significantly increase data interoperability between data providers, users, and applications.

#### Data cleaning

Data gaps and outlier values were identified and modified to generate the clean version of the time-series raw data. Multiple imputation methods via linear interpolation, K-nearest neighbors, and matrix factorization were proved to be effective when cleaning the time-series data of building electricity and HVAC operations in previous literatures^24,25^. Considering the length of data gap and the sampling frequency of each data point, three possible scenarios are identified as follows:

- ***Gaps that extend for no more than a few consecutive sampling frequencies (smaller gaps):*** In most cases these correspond to data gaps that are scattered through the dataset, which can be addressed by filling the gaps using *simple linear interpolation*.
- ***Gaps that extend for one hour up to a few hours or 1 day (small gaps):*** This kind of data gap lasts for a few hours and can be due to the sensors’ brief blackout. However, compared with the sampling frequency, data might be variously changed during these few hours. A simple linear regression is not sufficient to capture the dynamic pattern^25^. Therefore, we used more advanced linear interpolation, such as the *K-nearest neighbors* (KNN) algorithm. KNN is a generalization of the classic linear interpolation and is widely used in cases where relations among the dimensions of the data are complex. It imputes values using the weighted mean of the k most similar rows, weighted by their similarity.
- ***Gaps that extend from a few hours up to several days (large gaps):*** This is the case when a portion of the system or sensors is paused or faulted. In this dataset, the large gap only occurs for a subset of measurements and not the entire set of measurements; only the missing data corresponding to impacted measurements need to be dealt with to keep the data from the other measurements intact. Therefore, a more computationally intensive method is applied called *matrix factorization* (MF) to fill the large gap. MF was widely used to impute missing data. The algorithm assumes that different days of measurements (different rows of matrix) are generated from a shared subspace; thus, the data matrix of different days can be decomposed using a common factor.

Table 4 summarizes gap-filling strategies for smaller gaps, small gaps, and large gaps, based on the sampling frequency of each measurement. Normally, for data points with the sampling rate of 1 min, the upper thresholds for smaller gaps, small gaps and large gaps are set at 1 hour, 10 hours, and unlimited, respectively. For data points with the sampling rate of 5–15 minutes, upper thresholds are set at 10 hours, 1 day, and unlimited, respectively.

**Table 4:** Identification of outlier values and gap-filling strategies for all data points, and their missing rates.

Data	File name	Column name	Description	Number of data points	Unit	Sampling frequency	Missing rate for 3 years measurement	Specific available time period (if not Jan 2018–Dec 2020)	Gap filling strategy	Outlier criteria
Energy use data	ele.csv	mels_S	Miscellaneous electric load for the South Wing	1	kW	15 min	0.33		<10 hours: Linear 10 hours to 1 day: KNN >1 day: MF	<0
		mels_N	Miscellaneous electric load for the North Wing	1	kW	15 min	0.20			
		lig_S^+^	Lighting load for the South Wing	1	kW	15 min	0.18			
		hvac_S	Heating Ventilation and Air Conditioning load for the Sorth Wing	1	kW	15 min	0.08			
		hvac_N	Heating Ventilation and Air Conditioning load for the Nouth Wing	1	kW	15 min	0.08			
Outdoor environmental data	site_weather.csv	air_temp_set_1	Outdoor air temperature from sensor 1	1	°C	15 min	0.003			<0 °C or >50 °C
		air_temp_set_2	Outdoor air temperature from sensor 2	1	°C	15 min	0.005			
		dew_point_temperature	Outdoor air dew temperature of sensor 2	1	°C	15 min	0.011			
		relative_humidity_set_1	Outdoor air relative humidity from sensor 1	1	%	15 min	0.009			<0
		solar_radiation_set_1	Outdoor solar radiation from sensor 1	1	W/m^2^	15 min	0.009			
Indoor environmetnal data	zone_temp_sp_c.csv	zone_*_cooling_sp	Cooling temperature setpoint of Zone *	41	°F	5 min	0.05–0.07	Sep 2018–Dec 2020		<32°F or >122°F
	zone_temp_sp_h.csv	zone_*_heating_sp	Heating temperature setpoint of Zone *	41	°F	5 min	0.05–0.06			
	zone_temp_interior.csv	cerc_templogger_*	Zone temperature of interior zone	16	°F	10 min	0.01–0.21	Feb 2018–Dec 2020		
	zone_temp_exterior.csv	zone_*_temp	Zone temperature of exterior zone	51	°F	1 min	0.15–0.20		<1 hour: Linear 1 hour to 10 hours: KNN >10 hours: MF	
	zone_co2.csv	zone_*_co2	CO2 concentration of each zone	13	ppm	1 min	0–0.1	Aug–Dec 2019, Apr–Dec 2020		<0
HVAC operational data	hp_hws_temp.csv	hp_hws_temp	Heat pump heating water supply temperature	1	°F	1 min	0.14			<32°F or >122°F
	rtu_sa_t_sp.csv	rtu_*_sat_sp_tn	Roof Top Unit * supply air temperature setpoint (*: 001, 002, 003, 004)	4	°F	1 min	0.15			
	rtu_sa_t.csv	rtu_*_sa_temp	Roof Top Unit * supply air temperature (*: 001, 002, 003, 004)	4	°F	1 min	0.14			
	rtu_ra_t.csv	rtu_*_ra_temp	Roof Top Unit * return air temperature (*: 001, 002, 003, 004)	4	°F	1 min	0.14			
	rtu_ma_t.csv	rtu_*_ma_temp	Roof Top Unit * mixed air temperature (*: 001, 002, 003, 004)^++^	4	°F	1 min	0.14			
	rtu_oa_t.csv	rtu_*_oa_temp	Roof Top Unit * outdoor air temperature (*: 001, 002, 003, 004)	4	°F	1 min	0.14			
	rtu_sa_fr.csv	rtu_*_fltrd_sa_flow_tn	Roof Top Unit * filtered supply air flow rate (*: 001, 002, 003, 004)	4	CFM^+++^	1 min	0.14			<0
	rtu_oa_fr.csv	rtu_*_oa_flow_tn	Roof Top Unit * outdoor air flow rate (*: 001, 002, 003, 004)	4	CFM^+++^	1 min	0.02	Apr–Dec 2020		
	rtu_oa_damper.csv	rtu_*_oadmpr_pct	Roof Top Unit * outdoor air damper position (*: 001, 002, 003, 004)	4	%	1 min	0.15			
	rtu_econ_sp.csv	rtu_*_econ_stpt_tn	Roof Top Unit * economizer setpoint (*: 001, 002, 003, 004)	4	°F	1 min	0.14			<32°F or >122°F
	rtu_sa_p_sp.csv	rtu_*_pa_static_stpt_tn	Roof Top Unit * air pressure static setpoint (*: 001, 002, 003, 004)	4	psi^++++^	1 min	0.15			<0
	rtu_plenum_p.csv	rtu_*_fltrd_**_plenum_press_tn	Roof Top Unit * plenum air pressure at floor ** (*: 001, 002, 003, 004; **: gnd_lvl, lvl2)	8	psi^++++^	1 min	0.14			
	rtu_fan_spd.csv	rtu_*_sf_vfd_spd_fbk_tn	Roof Top Unit * supply fan speed (*: 001, 002, 003, 004)	4	%	1 min	0.14			
		rtu_*_rf_vfd_spd_fbk_tn	Roof Top Unit * return fan speed (*: 001, 002, 003, 004)	4	%	1 min	0.14			
	ashp_meter.csv	aru_001_power_mbtuph	Heat meter for air source heat pump	1	mbtuph^+++++^	5 min	0.29	Aug–Dec 2020	<10 hours: Linear 10 hours to 1 day: KNN >1 day: MF	
	ashp_cw.csv	aru_001_cws_temp	Evaporator/Cold water supply temperature	1	°F	5 min	0.01			<32°F or >122°F
		aru_001_cwr_temp	Evaporator/Cold water return temperature	1	°F	5 min	0.01			
		aru_001_cws_fr_gpm	Evaporator/Cold water fow rate	1	CFM^+++^	5 min	0.02			<0
	ashp_hw.csv	aru_001_hws_temp	Condenser/Hot water supply temperature	1	°F	5 min	0.16	Oct 2019–Dec 2020		<32°F or >212°F
		aru_001_hwr_temp	Condenser/Hot water return temperature	1	°F	5 min	0.16			
		aru_001_hws_fr_gpm	Condenser/Hot water fow rate	1	CFM^+++^	5 min	0.01			<0
	uft_fan_spd.csv	zone_*_fan_spd	Supply air fan speed of Zone *	44	%	1 min	0.15–0.23		<1 hour: Linear 1 hour to 10 hours: KNN >10 hours: MF	
	uft_hw_valve.csv	zone_*_hw_valve	Heating water valve position of Zone *	51	%	1 min	0.15–0.25			
Occupant data	occ.csv	occ_third_south	Occupant counts in the south half of third floor	1	/	1 min	0.0004	May 2018–Feb 2019		
		occ_fourth_south	Occupant counts in the south half of forth floor	1	/	1 min	0.0004			
	wifi.csv	wifi_first_south	Wifi connection counts in the south half of first floor	1	/	10 min	0	May–July 2018, Feb–Dec 2020	<10 hours: Linear 10 hours to 1 day: KNN >1 day: MF	
		wifi_second_south	Wifi connection counts in the south half of second floor	1	/	10 min	0			
		wifi_third_south	Wifi connection counts in the south half of third floor	1	/	10 min	0			
		wifi_fourth_south	Wifi connection counts in the south half of forth floor	1	/	10 min	0			

^+^No record for the lighting electricity in the north wing. Note that north and south wings are similar in both floor area and lighting systems.

^++^The Mixed Air Temp sensors on the RTUs were proven to be inaccurate due to poor installation and were replaced in early 2021.

^+++^1 CFM ~ 1.699 m^3^/h

^++++^1 psi ~ 6895 Pa

^+++++^1 btuph ~ 0.293 W

Table 4 summarizes outlier values following multiple criteria for different measurements. Generally, for electricity data, any value less than zero is considered an outlier value. For temperature data, any value less than 0 °C (32 °F) or larger than 50 °C (122 °F) is considered an outlier value, since Berkeley’s climate is considered to be mild. For other HVAC operational measurements (e.g., fan speed, air flow rate) and occupant measurements (e.g., occupant count, WiFi connected device count), any value less than zero is also considered as an outlier value. Since outlier values are all scattered for less than a few consecutive sampling frequencies through the dataset, the basic linear interpolation algorithm is applied to modify them.

After filling the data gaps and adjusting the outliers, the cleaned dataset has no more missing data.

## Data Records

As illustrated in Fig. 6, the dataset is organized in a three-layer pyramid structure. The final dataset is composed of the cleaned time-series operation data, the Brick model representing the metadata of the data measurements, and the JSON file representing the metadata of the dataset. The total size of the original data is about 2.38 GB (about 263 MB in a compressed file in zip format). The dataset is hosted at Dryad website^26^: 10.7941/D1N33Q.

### Time-series data

The time-series measured data collected from Building 59 can be organized into five major categories: energy use data, outdoor environmental data, indoor environmental data, HVAC operational data, and occupant data. After the data cleaning process, the entire dataset is compiled into 27 separate data files in the CSV format, containing 337 data points in total. Table 4 summarizes the available data points, as well as their locations in the data files, descriptions, sampling frequency, unit, and missing rate for the three-year measurement (specific available time period if applied).

### Metadata JSON File

A semantic metadata file is generated in the JSON format to represent the high-level data curation, contextual information, and application perspectives of the dataset. Four major aspects of the building and dataset information are summarized in the JSON file:

- Building information: information about the building and its service systems, including the geographic information, building, and systems characteristics
- Data governance: contextual information about the dataset, including the creation and curation logs of the dataset, as well as the sharing policy and contact information
- Data category: basic information about the available data points in 10 categories, including data format, data period, spatial and temporal resolution, as well as data quality indicators
- Applications: potential use cases of the dataset, reference publications, and access link.

### Brick model

**Fig. 7:**
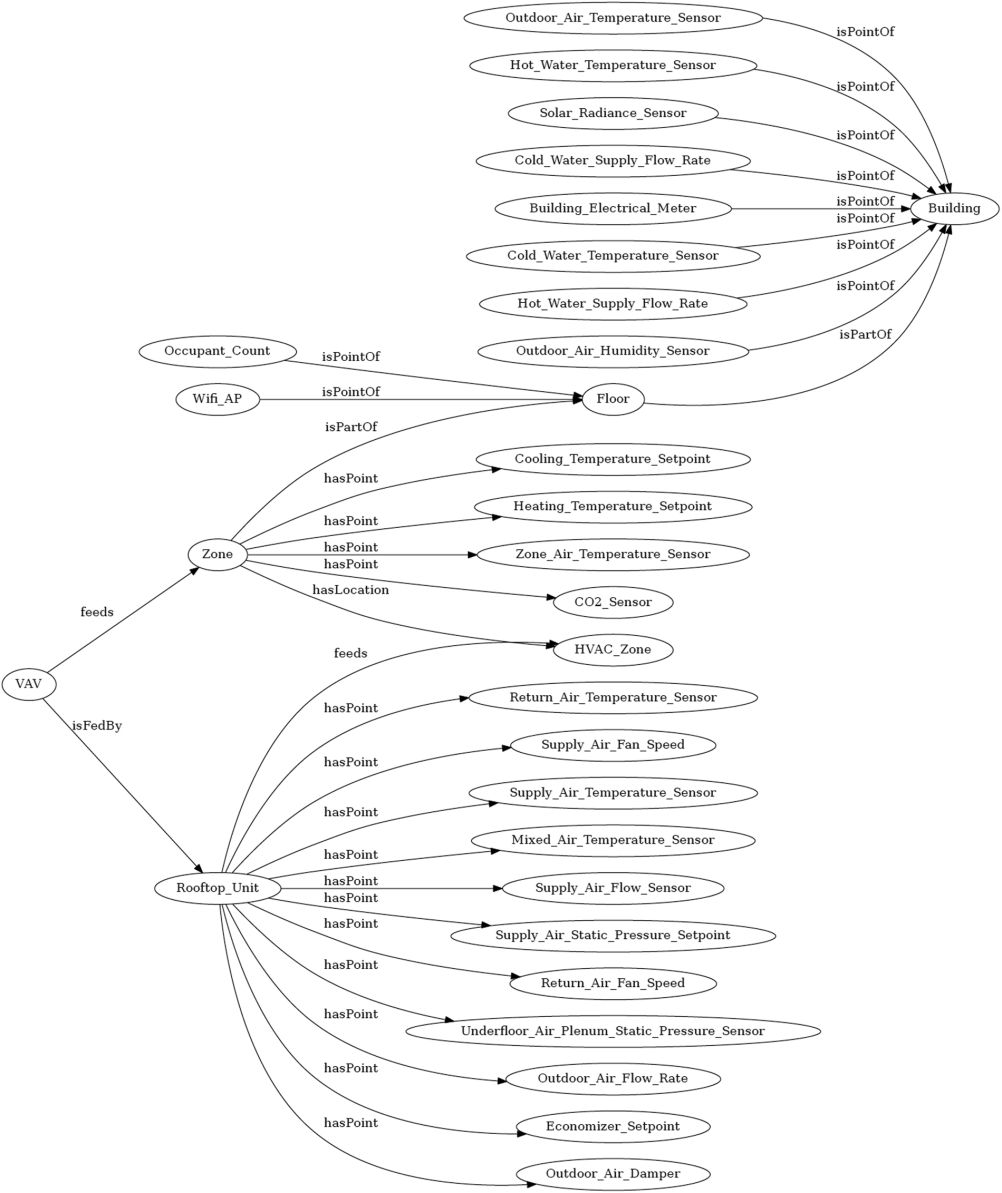
Illustration of the Brick model for the dataset.

In addition to the metadata from the whole building dataset, metadata from sensors and equipment is critical when using the dataset for further building operation analysis since it provides semantic information about the physical, logical, and virtual assets, as well information about their relationships in buildings. In this dataset, a Brick model represents the hierarchical structure of the building, systems, and sensors. Brick schema is an open-sourced data schema for standardizing semantic description of building assets^14^. It provides an extensible dictionary of terms and concepts, a set of relationships for linking and composing concepts together, and a flexible data model^27^ based on semantic web technologies. Figure 7 illustrates entity classes of the building and their relationships generated by the Brick TTL Viewer. Each entity has multiple instances with other entities. For example, the *zone* entity has a relationship with the *VAV* entity under the relationship of *feeds*; it also has relationships with indoor environmental sensors (e.g., *Zone_Air_Temperature_Sensor*, *CO2_Sensor*) under the relationship of *hasPoint*. The detailed Brick model is stored in the TTL file format.

## Technical Validation

### Whole-building energy use

Building 59 is an all-electric building, its energy use intensity (EUI) values of the two office floors were calculated for the three years (Table 5), and are compared with other office buildings in the state of California using the Building Performance Database (bpd.lbl.gov). The EUI of 2018 is higher than that in 2019 and 2020, which is due to the building retrofit for improving the building efficiency in 2019.

**Table 5:** EUI of the target building during the measurement periods.

C	2018	2019	2020
kJ/m^2^/year (SI unit)	465,617	317,983	340,696
kBtu/ft^2^/year (IP unit)	41	28	30

**Fig. 8:**
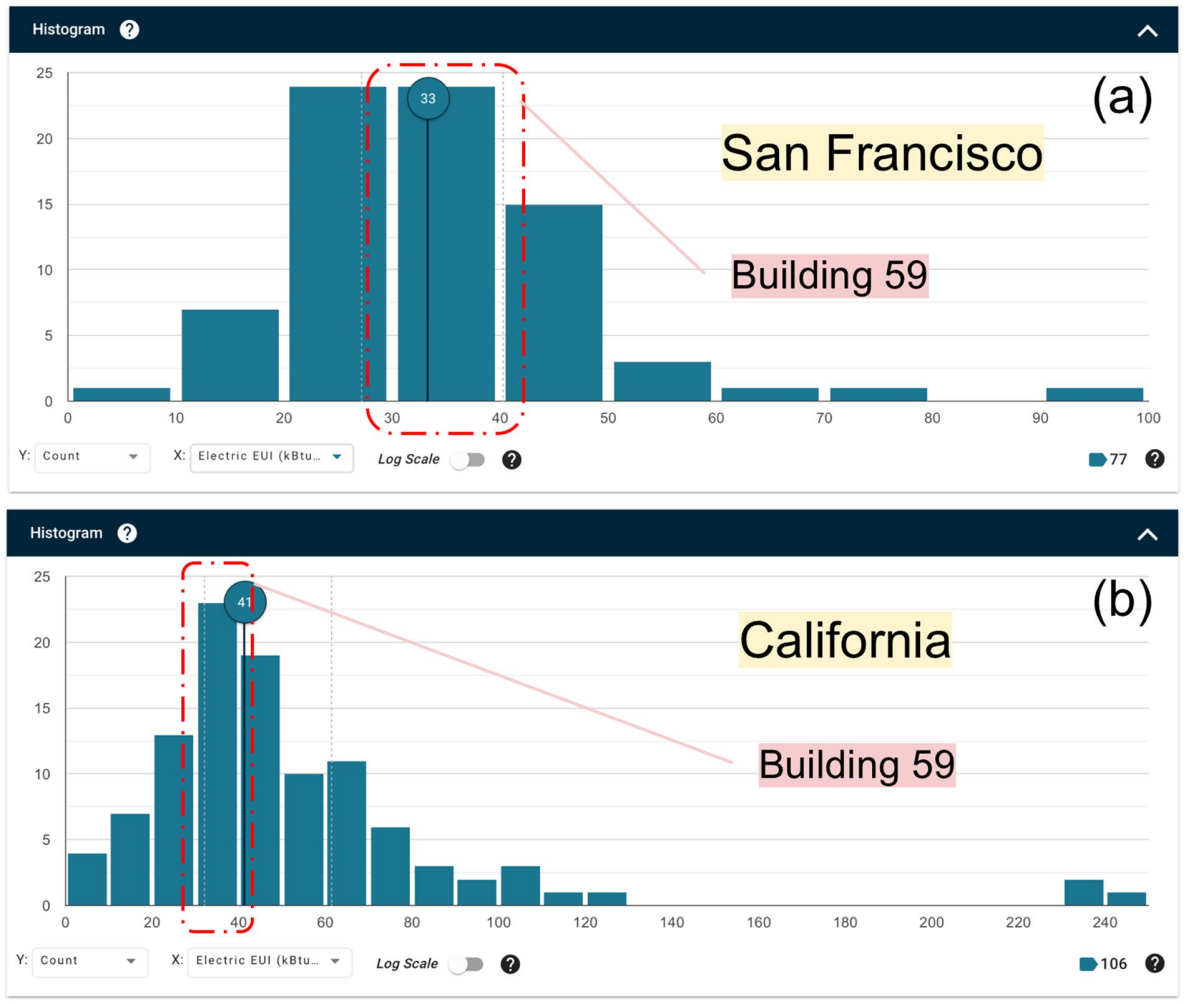
Electric EUI for San Francisco area (**a**) and state of California (**b**).

**Fig. 9:**
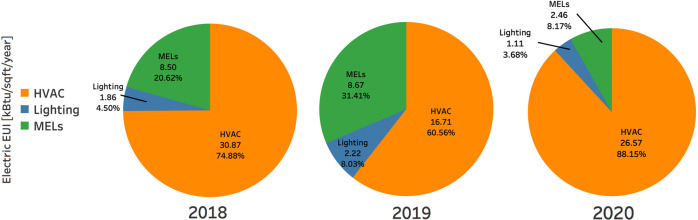
Electric EUI of major end uses from 2018 to 2020.

Figure 8(a) shows the histogram distribution of the available data points of the electric EUI for the San Francisco area. The data are selected from the Building Performance Database by filtering the building type as offices, year built within the past 30 years, and year when the data were collected within the past five years, using San Francisco Bay Area and the entire state of California, respectively as locations. The median value is 374,765 kJ/m^2^/year (33 kBtu/ft^2^/year), and the EUI of Building 59 belongs to the bin of data with highest frequency, from 227,131 to 454,261 kJ/m^2^/year (20 to 40 kBtu/ft^2^/year). Since the San Francisco area is generally cooler than the entire state of California, the EUI for the entire state is higher than that for San Francisco with a median value of 465,618 kJ/m^2^/year (41 kBtu/ft^2^/year), as shown in Fig. 9(b). Building 59 is still within the bin of data with highest frequency, from 340,696 to 454,261 kJ/m^2^/year (30 to 40 kBtu/ft^2^/year).

### Energy consumption disaggregation by end-use

Figure 9 shows three-year electric EUI for HVAC, lighting, and miscellaneous electric loads (MELs). Electricity consumed during HVAC operation accounts for 75%, 61%, and 88% of the total electricity consumption of the top two-floor area during 2018, 2019, and 2020, respectively. Lighting operations is the lowest energy consumer among end uses: less than 3% of the total electricity consumption.

**Fig. 10:**
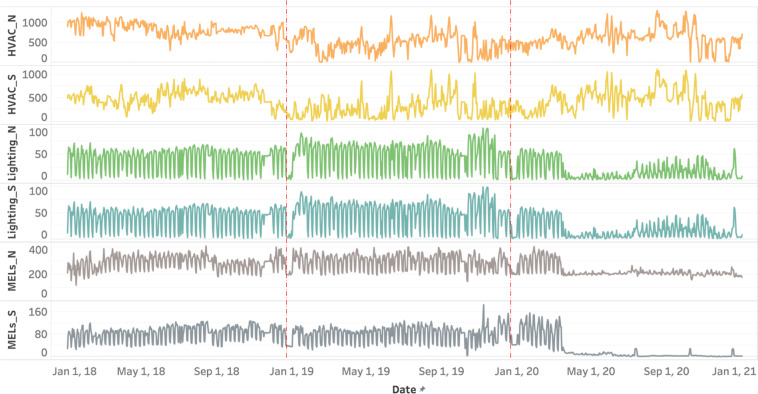
Time-series energy use (kWh/day) for major end uses from 2018 to 2020.

Figure 10 shows the time-series energy use that breaks down to these three major end uses. There is no significant reduction in HVAC electricity use during the pandemic, which is due to the higher requirement of ventilation air and the associated heating energy because of the cool climate in Berkeley. Lighting and MELs saw an electricity reduction of 50–85% starting in March 2020, at the first wave of the pandemic when the building had no occupancy. Lighting consumption started to increase in September 2020 as a limited number of people returned to the office gradually.

### Electrical load shape

**Fig. 11:**
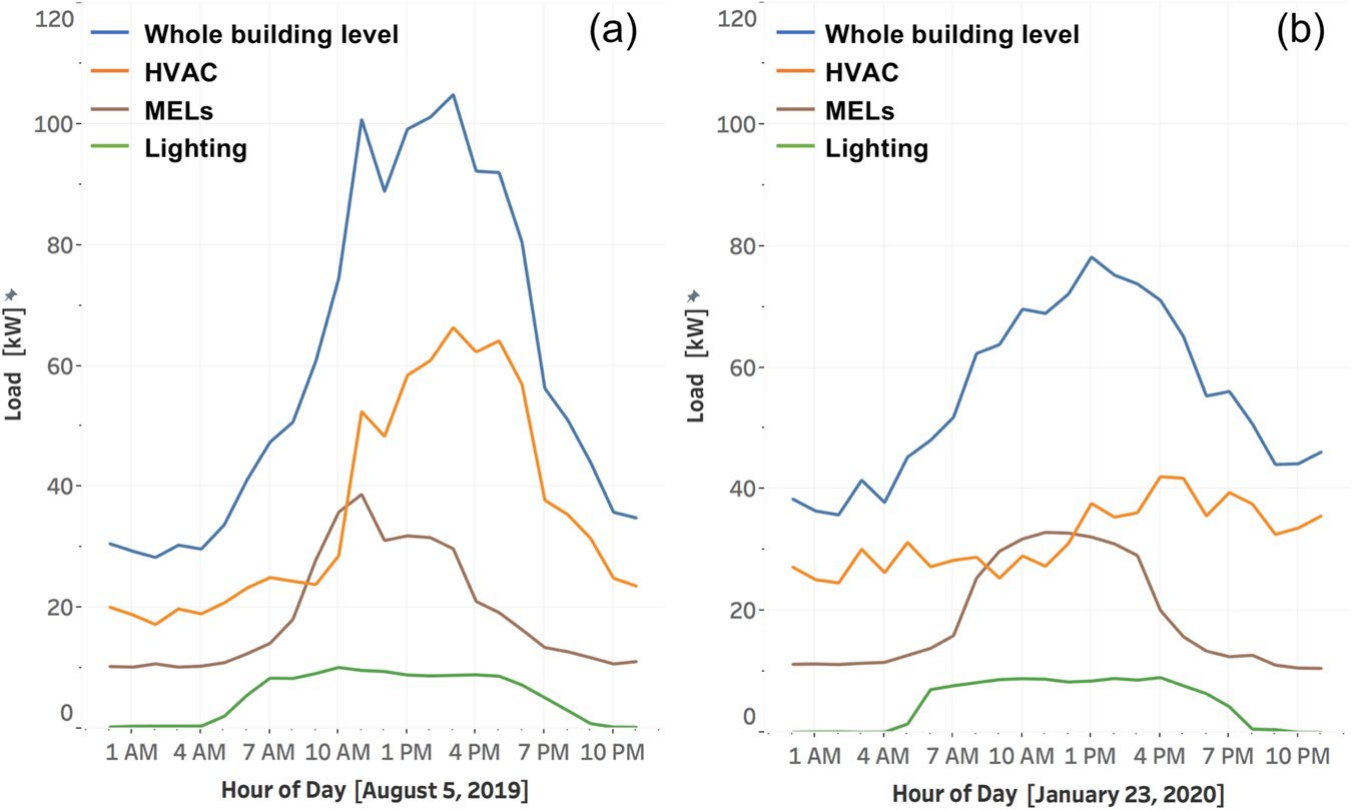
Load shape for both the whole building and major end uses on one typical summer day (**a**) and one typical winter day (**b**), before the pandemic.

A typical summer day (August 5, 2019) and a typical winter day (January 23, 2020) are used to analyze load shape before the pandemic for both the total and the major end uses of the top two floors, as shown in Fig. 11(a,b), respectively. In summer, the HVAC demand leaps around 10am, and remains high during the building operating hours until 6 pm. In winter, HVAC demand is relatively flat. MEL demand starts to increase around 7am, when occupants start to arrive in the office. MEL demand level is similar across summer and winter seasons. The lighting starts to consume more energy around 5am. Similar to MELs, lighting demand level is consistent across summer and winter seasons.

**Fig. 12:**
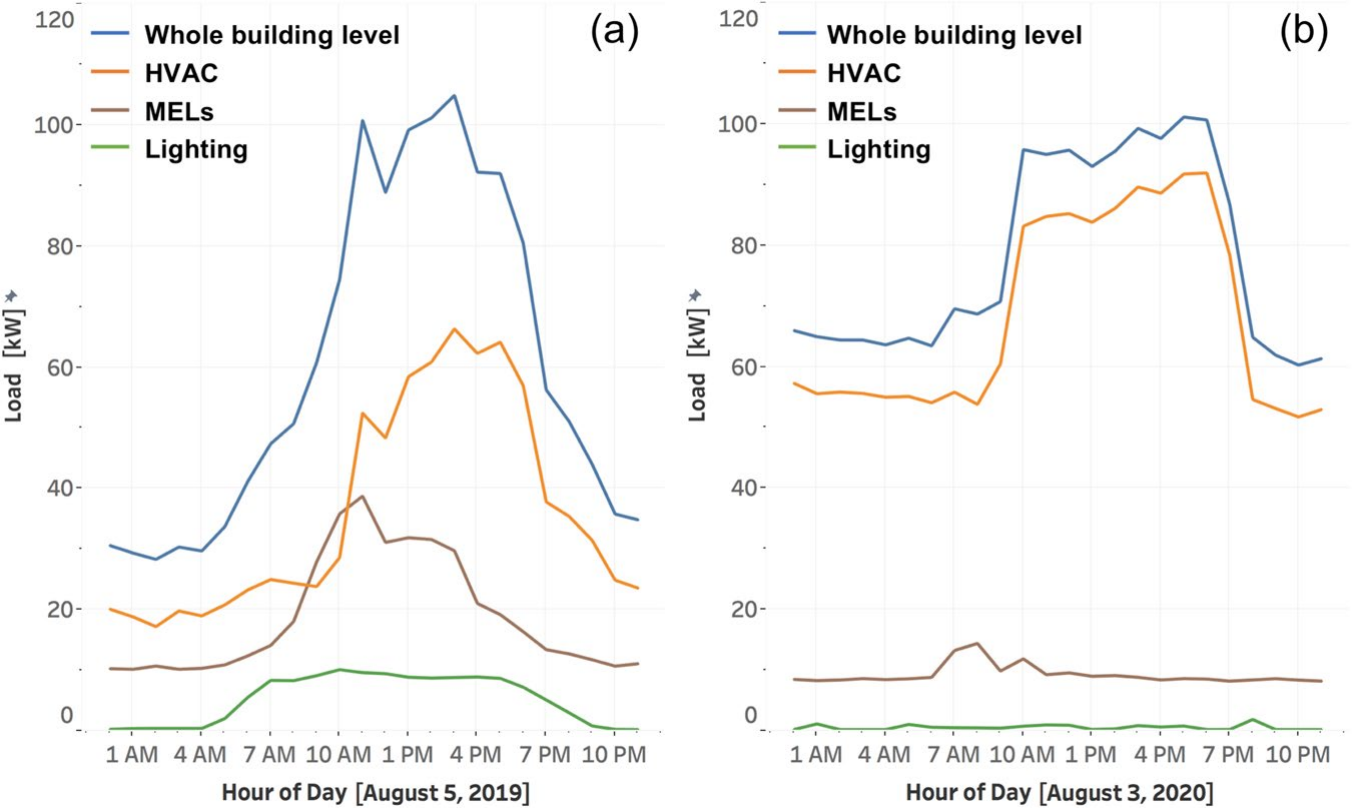
Load shape for both the whole building and end uses on one typical summer day before the pandemic (**a**) and one typical summer day during the pandemic (**b**).

Figure 12(a,b) show a comparison between load shape of a typical summer day before (August 5, 2019) and during (August 3, 2020) the pandemic. The comparison of HVAC load shape before and during the pandemic shows that HVAC consumed more energy during the pandemic, both during day and night, which is due to the higher requirement of ventilation air and the associated heating energy because of the cool climate in the San Francisco area. Lighting and MELs consumed much less energy during the pandemic since employees were mostly working remotely. Overall, the whole building level demand was similar to pre-pandemic levels.

### Floor map of the ground and second office floor

**Fig. 13:**
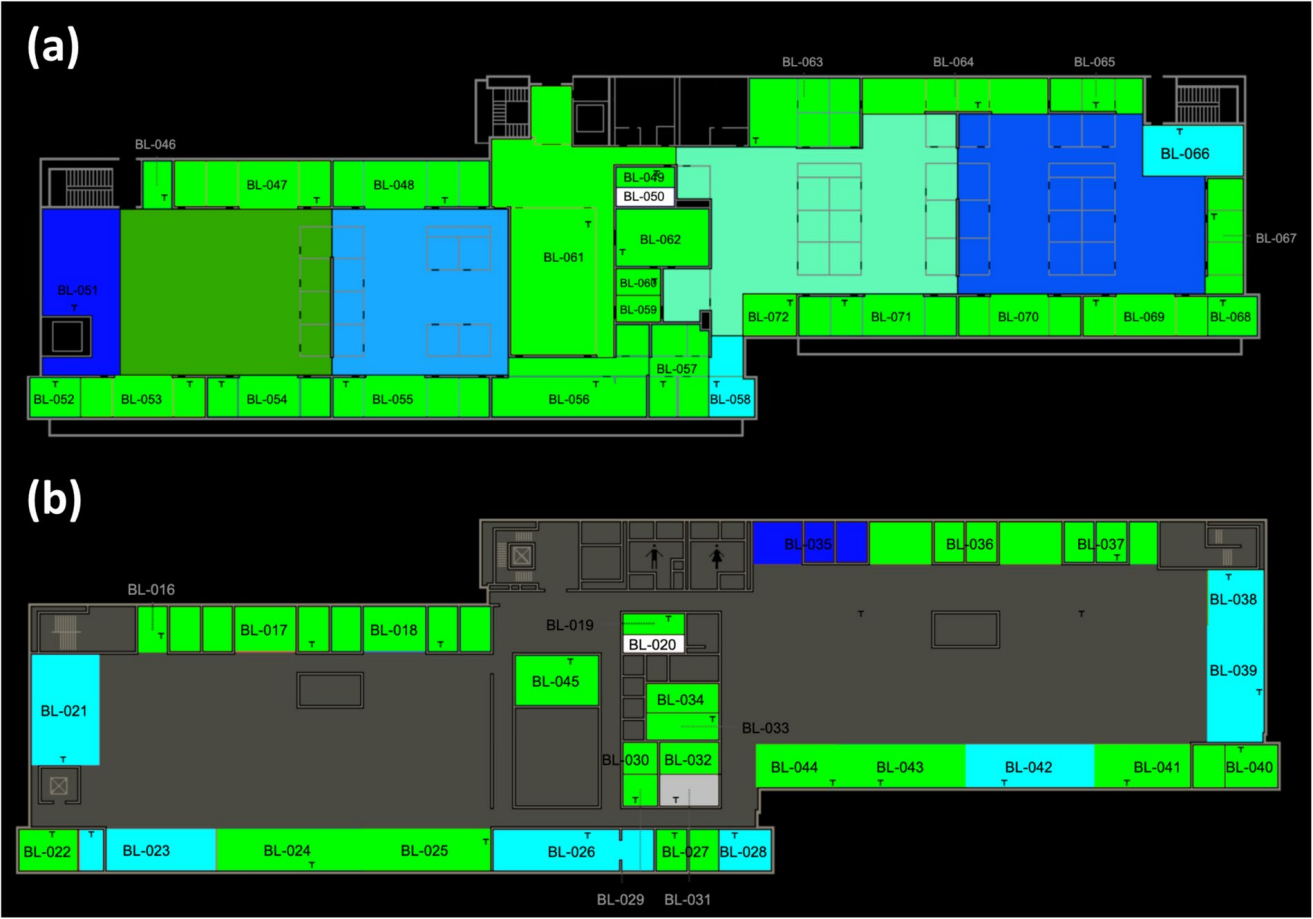
The floor map of (a) ground and (b) second floor about the location of each thermal zone.

To show the location of each thermal zone, we plot the floor map of the ground and second office floor in Fig. 13.

## Usage Notes

The time-series data are in CSV format and have a size of 2.38 GB. A more detailed note about the data cleaning strategy is available at the dataset’s GitHub page: https://github.com/LBNL-ETA/Data-Cleaning. An exploration of the metadata of equipment and sensors in the Brick model by using the Brick TTL viewer is recommended. Users can obtain high-level metadata about the building and dataset in the metadata JSON file.

## Data Availability

The Python code for detecting and filling the data gaps, as well as for modifying outlier values, is available at the dataset’s GitHub page: https://github.com/LBNL-ETA/Data-Cleaning.
